# Uncertainty Evaluation in Multistage Assembly Process Based on Enhanced OOPN

**DOI:** 10.3390/e20030164

**Published:** 2018-03-04

**Authors:** Yubing Huang, Wei Dai, Weiping Mou, Yu Zhao

**Affiliations:** 1 School of Reliability and System Engineering, Beihang University, Beijing 100000, China; 2 Quality Department, Luoyang Optoelectro Technology Development Center, Luoyang 471000, China

**Keywords:** assembly process, uncertainty evaluation, object-oriented petri net, defect stream, Shannon entropy

## Abstract

This study investigated the uncertainty of the multistage assembly process from the viewpoint of a stream of defects in the product assembly process. The vulnerable spots were analyzed and the fluctuations were controlled during this process. An uncertainty evaluation model was developed for the assembly process on the basis of an object-oriented Petri net (OOPN) by replacing its transition function with a fitted defect changing function. The definition of entropy in physics was applied to characterize the uncertainty of the model in evaluating the assembly process. The uncertainty was then measured as the entropy of the semi-Markov chain, which could be used to calculate the uncertainty of a specific subset of places, as well as the entire process. The OOPN model could correspond to the Markov process because its reachable token can be directly mapped to the Markov process. Using the steady-state probability combined with the uncertainty evaluation, the vulnerable spots in the assembly process were identified and a scanning test program was proposed to improve the quality of the assembly process. Finally, this work analyzed the assembly process on the basis of the uncertainty of the assembly structure and the variables of the assembly process. Finally, the case of a certain product assembly process was analyzed to test the advantages of this method.

## 1. Introduction

In manufacturing mechanical products, product quality is directly determined by the assembly process quality due to the great variety of components and the complexity of the structures involved. A multistage assembly system, which consists of multiple components that must be finished within a given time [1] is widely used in the automotive, ship-building, and appliance industries. The assembly process is found at the end of the manufacturing cycle; if the quality defects are not eliminated in the assembly process, the product delivery cycle and customer satisfaction are directly affected [2]. To meet this need, the object of quality control should be shifted from the final product to the process itself. The modern assembly process is the product of multiple composite cross-disciplines with perfect control function, complex structure, and high automation degree, and within such a system, the potential for the introduction of defects is heightened. At present, the manufacturing process is mainly controlled and analyzed by building a model that uses the Markov chain or Petri net [3] using the Markov chain to model the aircraft assembly process in an idealized way. Meanwhile Petri nets are a good graphical tool for analyzing system behavior via state equations, algebraic equations, and other mathematical methods, which has been used in modelling the manufacturing system, network configuration, etc. Therefore, Petri nets provide a good approach to evaluate the uncertainty of the assembly process. In the reliability analysis of computer systems, models, such as fault trees, Markov chains, and stochastic Petri nets, are built to evaluate or predict the reliability of the system [4]. However, most Petri nets are applied to path-planning problems, such as scheduling systems or to the analysis of assembly efficiency through component networks. Few studies have been conducted on the impact of assembly networks on product quality. Therefore, the variation of residual stress in the assembly process was combined with the assembly Petri net, analysis of the assembly model in the determination of the process and technology of entropy to characterize the uncertainty of the model, in order to assess the quality of assembly. 

Zhou and Yuan [5] combined the analytic hierarchy process and Petri net to evaluate the assembly process in a qualitative way, and Gao and Wen [6] continued to emulate and optimize the assembly process. Using performance analysis Jahanzaib [7] improved the performance parameters of the assembly process to increase the quality. Based on the basic Petri net, Bohez [8] analyzed optimization sequence and performance based on Petri nets in flexible assembly process. The above research is mainly aimed at the influence of single process, without considering the influence of residual stress after multi-process coupling. Zhang [9] and Qian [10] used the fuzzy method to measure the uncertainty of the assembly process. Yianni and Rama attempted to model railway bridge deterioration, as well as the inspection and intervention processes to provide a more rounded overview of railway bridge asset management [11]. In the assembly process, from the part level to the component level, and then to the system level, the introduction and release of residual stress follows. Franciosa proposed a methodology for the rapid diagnosis of defects generated in a single-stage process and propagated through the multistage assembly system [1]. Therefore, the uncertainty of the multistage assembly process is analyzed according to the trend of defect change.

Depending on the size and location, each defect in the device affects reliability. A fatal or killer defect is one that is of sufficient size and occurs in a place where the outcome is an immediate device failure [12]. For example, when the product size exceeds the design threshold after processing; such a defect is detected after the manufacturing or screening test. Meanwhile, a defect that is either too small or is located in a position that does not cause an immediate failure is called the latent defect. A latent defect may or may not cause failure in the field, depending on the operating time, environmental condition, and processes. In order to understand the evolution of the potential defects, further analysis of the dominant influence on the reliability of the product, must be conducted. Doing so can help determine the deal assembly sequence and screening position. Previously, some attempts have been made to extend defect models to estimate reliability. At first, a simple time-independent Poisson reliability was obtained assuming directly that the number of nonfatal defects corresponded to the fatal defects following a Poisson distribution [13,14,15]. All models implicitly assumed that the number of fatal defects is independent of the number of the latent defects in a device. From the multinomial distribution, the number of fatal defects and the number of nonfatal defects in a device are negatively correlated if the total number of defects in a device is fixed. 

Uncertainty can be defined as the lack of precise knowledge as to what the qualitative or quantitative truth is. Uncertainty is ubiquitous in nature, and finding ways to measure is has attracted much attention [16]. Numerous uncertainty theories have been developed [17], such as probability theory, fuzzy set theory, possibility theory, and defect numbers. Barchielli and Deng evaluated the uncertainty of the data by using different forms of entropy [18,19,20]. In model evaluation, Zeng and Wu [21] evaluated the mean uncertainty of the Bias model by the Shannon entropy; Ibl and Capek [22] measured the uncertainty in the SPN (Stochastic Petri Net). However, this kind of research is still in the stage of theoretical analysis, and only a few engineering applications have been used. Quantitative uncertainty analysis is not well explored. The uncertainty analysis of the system is based on the given assembly process. With the random variables associated with the Petri net transitions, the dynamic behavior of the cooperating satellites in a SPN model can be mapped onto a time-continuous Markov chain with discrete state space. Once a Markov SPN model is generated, the probability of a given condition in the network at a specified time can be computed and quantified, along with the vulnerability and uncertainty of the system using the identified indicators [23]. The component model is then developed by combining the assembly process structure with the latent defect caused by the assembly process. Uncertainty is measured as the entropy of the semi-Markov chain because calculating the uncertainty of a specific subset of sites as well as the entire network is now possible [24]. The entropy of the stochastic event Petri net is used to calculate the entropy of the model when time follows a certain distribution, thus the uncertainty of the assembly process is evaluated. 

In the next section, an improved Petri net was developed to describe the assembly process considering the change of residual stress during this process. Then, the uncertainty of the improved Petri net was evaluated and the vulnerable points were located. Finally, an example related to unmanned aerial vehicle (UAV) was analyzed using the proposed model. 

## 2. Assembly Process Model Based on Petri Net and Defect Analysis

Petri nets are a graphical and mathematical modelling notation first introduced by Carl Adam Petri’s dissertation published in 1962 at the Technical University Darmstadt. A Petri net consists of places, transitions, and arcs that connect them. Places are drawn as circles, transitions as rectangles, and arcs as arrows. Input arcs connect places with transitions, and output arcs connect transitions with places. Places are passive components and model the system state. They can contain tokens, depicted as black dots or numbers. The current state of the Petri net is given by the number of tokens at each place. Transitions are active components that model activities that can occur; a change of state can take place with the assignment of new tokens to places. Transitions are only allowed to occur if they are enabled, which means that at least one token is available on each input place. By occurring, the transition removes a token from each input place and adds a token to each output place. 

There are three general characteristics of Petri nets that make them interesting in capturing concurrent object-oriented behavioral specifications. First, Petri nets allow the modeling of concurrency, synchronization, and resource sharing behavior of a system. Second, Petri nets strictly distinguish the activity from the implementation, the expression ability is richer, and the flexible characteristic is more obvious. Finally, many theoretical results associated with Petri nets are available for the analysis of several issues, such as deadlock detection and performance analysis [25,26,27]. The Petri net is a tool for the representation and modeling of dynamic systems [8]. The assembly process modeling is applied to represent the relationships among them, to describe the elements of the performance and system assembly process, and to reveal the relationship between them. Thus, the assembly process must be analyzed, optimized, and controlled. According to Discrete Event System (DES), systems can be represented as events and states, along with the relationships that exist among them. The assembly process can be considered as an organic whole of assembly-related tasks or processes. These tasks and processes can be expressed abstractly in a series of events.

### 2.1. Structure of the OOPN

The assembly process consists of the assemblies of parts, components and complex systems. The object-oriented Petri net (OOPN) model is designed to facilitate the simulation of the assembly process reliability and the analysis of the complex system. In applying the concept of “object” and “message passing” in the object-oriented method (OOM), the OOPN uses subnets to encapsulate the internal behaviors of an object and broadcast places to transmit the shared information without time delay. A transition function is used in depict logics and arcs linking subnets and state transitions. The assembly system consists of several objects, each of which possesses the behavior that is represented by the method, as well as the attribute or state. The object carries on the corresponding activity according to its input information, and the information transfer between the object controls the activity and sequence of the different objects. The information transfer between objects is described by changes and the network with a directed arc. The Message Passing Relation net (MPRN) is a mathematical theory and model that is best used with the OOPN as they can simplify the model by focusing on the information transfer among objects and the relationship between object and outside world. The excitation function of defects in the assembly process refers to the transmission or reception mechanism between objects.

By replacing its transition function π with the latent defect transform in the dominant defect, the OOPN represents the transform’s rate of change, whose value is the reciprocal of the change correction of the latent defect. When the assembly fails, the threshold value of h equal to the present value.

The definition of the OOPN is:

(1) $$OOPN=(\mathrm{OP},\mathrm{OT},\mathrm{OF},D,Q,\pi ,H,M_{0})$$

In OP=P∪R∪IM∪OM. OP refers to the set of places; P is the state set in objects; R represents the source set in objects; IM stands for the set of input information; and OM is the output information set among objects.

In OT=T∪G. OT is the transform set. T refers to the transform set in object and G stands for the set of transforms among objects.

In OF=F∪DF, wherein F represents the front and back relations of the assembly process for the directed arcs of the same level of process, DF refers to the arcs set in different levels.

In ${D=D}_{p}\cup D_{t}\cup D_{f}$, D refers to the defect change, Q is a set of all subnets, and each subnet is an OOPN.

π: This activation function represents the defect stream function which presents the probability of the latent defect being transformed into a dominant defect over time. The set of excitation rates is *λ_0_* = {*λ_i_*}. This function submits to the hypoexponential distribution.

H: This is a constant representing the threshold of defect calculated by the cumulative risk. If the threshold is exceeded, the excitation function fails and assembly stops. 

*M*_0_: This is the initial value, which is a Boolean value. If the value of *M*_0_ is 1, this means the node starts working; otherwise, it stops working if the value is 0.

### 2.2. Transition Function with the Defect Analysis

Latent defects and dominant defects exist in the probabilistic assembly process, in which the time potential defects are transformed, turning into dominant defects. If the latter is activated, then the product fails, and the next assembly stops. Considering the definition, the potential defects and dominant defects in the assembly process are subject to exponential distribution. The time hypoexponential distribution of the whole assembly process defect change is expressed below.

Where two parameters in the distribution are present ($\rho_{1}\neq \rho_{2}$), the explicit forms of the probability function and the associated statistics are given by:

(2) $$F(x)=1-\frac{\rho_{2}}{\rho_{2}-\rho_{1}}e^{-\rho_{1}x}+\frac{\rho_{1}}{\rho_{2}-\rho_{1}}e^{-\rho_{2}x}$$

(3) $$f(x)=\frac{\rho_{2}\rho_{1}}{\rho_{1}-\rho_{2}}(e^{-\rho_{2}x}-e^{-\rho_{1}x}),x>0$$

The variation coefficient is always <1. With the sample mean and sample coefficient of variation (c), the parameters $\rho_{1}$ and $\rho_{2}$ can be estimated as follows, the sample mean, and sample coefficient of variation are collected from the history data:

(4) $$\rho_{1}=\frac{2}{x}{\left[1+\sqrt{1+2(c^{2}-1)}\right]}^{-1}=0.87$$

(5) $$\rho_{2}=\frac{2}{x}{\left[1+\sqrt{1+2(c^{2}-1)}\right]}^{-1}=3.2$$

The resulting parameters $\rho_{1}$ and $\rho_{2}$ are real values if $c^{2}\in \left[0.5,1\right]$. The efficiency of this distribution is given by:

(6) $$h(t)=\frac{\rho_{1}\rho_{2}(e^{-\rho_{2}x}-e^{-\rho_{1}x})}{\rho_{1}e^{-\rho_{2}x}-\rho_{2}e^{-\rho_{1}x}}$$

(7) π=f(x)

In this paper, the assembly process of the board component is studied, and the life of trigger parts is evaluated by adopting the two-parameter Weibull distribution [24]:

(8) $$F(t)=1-e^{\left(-mt\alpha \right)}$$

(9) $$f(t)=m\alpha t^{\alpha -1}e^{-mt\alpha }$$

Using cumulative risk to define the threshold of each process, we have:

(10) $$H(t)=mt^{\alpha }$$

Due to the different parts, slight fluctuations may occur in the function. Therefore, after adjusting the relative index, the accuracy of the guarantee function is given by:

(11) $$\bar{R_{k}^{2}}=1-[\frac{n-1}{n-k}(1-R_{k}^{2})]$$

The optimized function *F*(*x*) is presented below:

Shape parameter *m* = 1–42306803953;
Scale parameter α = 45.9183276528; and
Correlation coefficient = 0.971886604314.

### 2.3. Semi-Markov Chain

In this section, a method is proposed to obtain the performance parameters from the OOPN in order to analyze the stochastic behavior of the system. The embedded continuous-time Markov chain (CTMC) is derived from the OOPN, and the Markov chain theory is adopted to obtain the performance parameters.

SPN is a time Petri net, when random variables are utilized to specify the time behavior. Under certain conditions, SPNs are isomorphic to homogeneous Markov chains. According to the analysis on the metrics of the Markov chain (such as the steady state probability distribution), investigating the behavior of the underlying system being modeled by the Petri net is possible. The activity diagram is translated to the OOPN model by employing the transformation algorithm.

The stochastic process associates the OOPN systems with M_0_, which can be classified as a finite state space, stationary, irreducible, and continuous-time semi-Markov process. In the case of the OOPNs, the embedded Markov chain can be recognized disregarding the concept of time and focusing on the set of states of the semi-Markov process. The specifications of an OOPN system are sufficient to calculate the transition probabilities of such a chain. If the change function on the OOPN is a random variable of exponential distribution, the model can be transformed into a random process with Markov characteristics. Based on the analysis of in Section 2.2, the change of state of the assembly process is subject to hypoexponential distribution. In the semi-Markov process, the time of state transfer can be randomly distributed. Therefore, the semi-Markov process should be utilized to address this problem.

Supposing the state of the system in *M* states is *t*, *M*{1,2,…,*M*}. and the initial state of the system is *i*, when *t* = 0 the single-step transition is:

(12) $$G(0)=i,i\in \left\{1,2,\ldots ,\in M\right\};\pi_{ij}=P\left\{G(t_{m})=j|G(t_{m-1})=i\right\},i,j\in \left\{1,\ldots ,K\right\}$$

where $\pi_{ij}$ is the probability ranging from state *i* to state *j*, which determines the single-step transition probability matrix *G*(*t_m_*). Given that the type of *t_m_* is a discrete value the *G*(*t_m_*) is the embedded Markov chain. As time goes on, the defects in the system can cause the product to fail, and the state of the system gradually degrades. Assuming the time *T_ij_* in state *i* conforms to the distribution of *F_ij_*(*t*), when $\pi_{ij}\neq 0$, $F_{ij}(t)=F_{ij}(T_{ij}\leq t)$. *F_ij_* is the probability density function of $\pi_{ij}$, *T_ij_* represents the time stayed at the state *i* before transforming to state *j.* In this paper, *F* refers to the probability of the latent defect being activated as a dominant defect over time. If *F_ij_*(*t*) accords with exponential distribution, the system can be described in Markov process. The kernel matrix *Q*(*t*) is obtained by the competitive behavior between the failure distribution and the state transfer matrix. In every element of *Q*(*t*), *Q_ij_*(*t*) means the single-step transition probability from state *i* to state *j* in [0,*T*]. For a multi-state system that is subject to a semi-Markov state, the dynamic characteristics of the system can be defined if the initial state vector *P*(0) and the kernel matrix *Q*(*t*) are maintained. The set $\theta_{ij}(t)$ stands for the probability of the state of the system, when *t* = 0, the state is *i* and when at *t*, the state is *j*:

(13) $$\theta_{ij}(t)=\delta_{ij}[1-F_{i}(t)]+\sum_{k\in M}\int_{0}^{t}\theta_{kj}(t-x)dQ_{ik}(x),i,j\in M$$

Among them:

(14) $$q_{ik}(t)=\frac{dQ_{ik}(t)}{dt}$$

(15) $$F_{i}(t)=\sum_{j=1}^{k}Q_{ij}(t)$$

(16) $$\sigma_{ij}=\{\begin{array}{c} 1,if\;i=j\\ 0,if\;i\neq j \end{array}$$

Calculating the probability of states *P*(*t*) by the transition probability matrix $\theta_{ij}(t)$, we have:

(17) P(t)=P(0)⋅θ(t)

The assembly process based on OOPN is a top-down modeling, which can be divided into the following steps [18] described below:

1. The OOPN is proposed by representing its transitions with the fitted latent defect stream function. Step 1–4 describe the process of building the module of the OOPN:
  - Step 1: Create assembly object sets *ob* = {*ob1,ob2…,obn*}, considering that different assembly units are separate assembly objects.
  - Step 2: *G* is the transition among objects. According to the assembly flow chart, the transition in the information transfer of each object is inserted at the beginning and at the end of the object. *G* = {*g1,g2,…,gn*}.
  - Step 3: Aimed to each object Ob, IM is the input information place and the OM, the output information place has been marked. The object inside is made up of the basic Petri net *N* = (*P, T, F*).
  - Step 4: Create *F*, which is the set of arcs of the states.
2. *M*_0_ is the initial value, the same as the definition in the OOPN.
3. Set of all reachable marking
  Let OOPN be an object-oriented Petri net. The set of all reachable marking from initial marking *M*_0_ in OOPN is denoted by *R*(*M*_0_), which represents the reachable marking:
  (18) $$R(M_{0})=\left[\begin{array}{cccc} M_{0}(P_{1}) & M_{1}(P_{1}) & \cdots & M_{\left[R(M_{0})\right]}(P_{1})\\ M_{0}(P_{2}) & M_{1}(P_{2}) & \cdots & M_{\left[R(M_{0})\right]}(P_{2})\\ \vdots & \vdots & \ddots & \vdots \\ M_{0}(P_{m}) & M_{1}(P_{m}) & \cdots & M_{\left[R(M_{0})\right]}(P_{m}) \end{array}\right]$$

Each arc in the graph provides the excitation rate of the corresponding change of the arc, thus obtaining the semi-Markov chain (the excitation rate of this paper is related to the identification). The change of the arc refers to the probability of the density function showing defects in the changing process from latent to dominant. The OOPN is developed from the SPN, so it can transform to the semi-Markov chain by isomorphism.

## 3. Uncertainty Evaluation of Assembly Process

### 3.1. Uncertainty and Shannon Entropy

The concept of entropy is derived from thermodynamics. In 1948, Shannon introduced the concept of information entropy, and in 1977, Boltzmann introduced the statistical entropy.

Entropy refers to the degree of confusion within the system [14]. If entropy is 0, the molecules inside the system is are uniformly distributed. Converted to an assembly process, if its entropy is 0, the parameters and results of each step in the assembly process are even. It can be imagined that the system of an assembly process is the same without affecting each other, and must be modest. Accordingly, if the entropy close to 1, the more chaotic the system is, and the poorer controllability the system will have. The calculation of uncertainty characterizes not only the risk of the assembly process, but also its characterize the complexity, which is conducive to making better decisions.

As an efficient tool, Shannon entropy is used to measure the uncertain information. The definition of uncertainty in the Petri net is given by:

Uncertainty = Entropy/Log_2_|*R*(*M*_0_)|

where *R*(*M*_0_) represents the reachable marking.

### 3.2. Uncertainty Calculation

The following analysis describe herein is conducted for the aforementioned Petri net model:

According to the possibility of representing the set of all reachable markings while considering the semi-Markov chains, defining the transition rate matrix must be done to meet the requirement of defining the stationary probabilities of all marking.

1. Transition rate matrix
  Let the OOPN be an object-oriented Petri net. The transition rate matrix *Q* of OOPN is defined as:
  (19) $$Q:(R(M_{0})\times R(M_{0}))\to R$$
2. Steady-state probability
  In this semi-Markov chain, the excitation rate is associated with the identification. The steady-state distribution vector *μ* is defined as the normalized left null space of transition matrix *Q*:
  (20) $$\mu Q=0,\mu 1^{T}=1$$
  Vector *μ* represents the steady-state probability of each OOPN marking:
  (21) $$\mu =\left[\begin{array}{c} \mathrm{Pr}(M_{0})\\ \mathrm{Pr}(M_{1})\\ \vdots \\ \mathrm{Pr}(M_{\left|R(M_{0})\right|}) \end{array}\right]$$
  The long-term probability of marking $M\in R\left(M_{0}\right)$ is defined as a corresponding element if vector *μ* is:
  (22) $$\mu_{i}=\mathrm{Pr}(M_{i})$$
  The probability of marking can be regarded as a joined probability of individual places in a specific marking.
  (23) $$\begin{array}{l} \mathrm{Pr}(M)=\mathrm{Pr}(M(p_{1}))=x_{1},\\ M(p_{2})=x_{2},\ldots ,M(p_{m})=x_{m} \end{array}$$

In the calculation of steady-state probabilities, the liveliness of the Petri net must be appropriate as each dead marking corresponds to an absorbing state in the semi-Markov chain. The assembly process is a system with limited resource allocation. The main consideration in this paper is the probability of excited dominant defects that can be subjected to the process. If the cumulative risk is too high, conflict can be caused in the Petri net. As a result, it can be utilized to determine whether there is a conflict before uncertainty calculating. Every absorbing state can occur, and its stationary probability is equal to 1, hence, all live markings have stationary probabilities that are equal to 0, which would lead to a fully-deterministic model without any uncertainty. 

Hence, using entropy can measure the amount of disorder (uncertainty) that is associated with a random variable.

3. The entropy of the random variable X is defined as:
  (24) $$H(X)=-\sum_{x}\mathrm{Pr}(X=x)\log_{2}\mathrm{Pr}(X=x)$$
  In Equation (24), H will reach its maximum value if all states are equiprobable, that is, if an indication of an assumption that all states have equal probability. Like variety, *H* expresses our uncertainty or ignorance of the state of system. *H* = 0 can be represented, if and only if the probability of a certain state is equal to 1 and all other states are equal to 0 [28,29]. In that case, we will obtain maximum certainty or complete information of the system in. which it is found. A constraint that reduces uncertainty is defined, as the difference between the maximum and actual uncertainty, which can also be interpreted in a different way. Indeed, if some information about the state of system is acquired, our uncertainty about the state will decrease by excluding or reducing the probability of a number of states [30]. The information acquired from an observation is equal to the degree to which uncertainty is reduced.
4. Entropy of the stochastic Petri net.
  *μ* is the vector of its stationary probability:
  (25) $$\mu_{i}=\mathrm{Pr}(M_{i}),M_{i}\in R(M_{0})$$
  The entropy of the OOPN is defined as:
  (26) $$H(OOPN)=-\sum_{i=1}^{\left|R(M_{0})\right|}\mu_{i}\log_{2}\mu_{i}$$
5. Uncertainty index of stochastic Petri net.
  *H* represents the entropy. The uncertainty index of OOPN is defined as:
  (27) $$UI(OOPN)=\frac{H(OOPN)}{\log_{2}|R(M_{0})|}$$
  The uncertainty index ranging in (0,1) is calculated. Wherein, 0 interprets the full deterministic model and 1 interprets the absolute chaotic model. When the value of uncertainty index is closer to 1, the behavior of the model will be less predictable.

### 3.3. Ensuring the Vulnerable Points in the Assembly Process

Previous analyses of the assembly process did not consider the test link between processes. However, in the actual project, increasing tests are required in order to enhance the quality of the product. In this case, determining the location of detection by analyzing vulnerable points is helpful because point-by-point detection is not feasible. In this paper, a vulnerable point is defined as the point that is uncertain and can be improved after testing. First, the objects are ranked from large to small with the number of steady-state probability values calculated in Section 3.2. Second, based on the order, the analysis of the test link should be added to the uncertainty degree of the assembly process, in which n times must be analyzed if n objects exist. Finally, the correct number of test points is selected for the assembly process testing based on the actual working conditions.

## 4. Case Study

**Figure 1:**
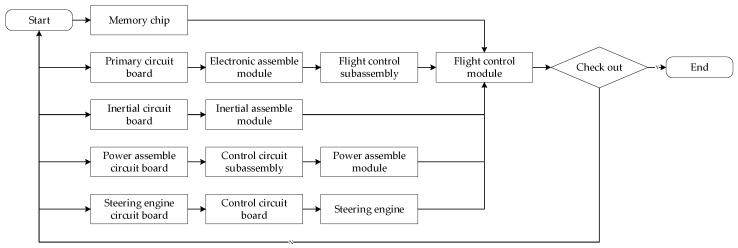
The flowchart of the assembly process of a flight control module.

At present, UAVs are now more popular and play an increasingly significant role in various fields. As they are now being customized, the assembly process of a UAV occupies over 50% of the total workload. The assembly process of the flight control of UAVs are taken as an example and analyzed, and the flowchart of the assembly process is shown in Figure 1.

From left to right, we can see the parts and components, respectively. As can be seen, the entire assembly process is a multi-concurrent structure. Due to the small size and highly precise workpieces, the sensitivity of the defect stream in the assembly process is enhanced. Therefore, the uncertainty of the assembly process can be evaluated. The assembly process analysis is conducted by replacing the activation function of the Petri net with the defect stream introduced by the assembly process.

**Figure 2:**
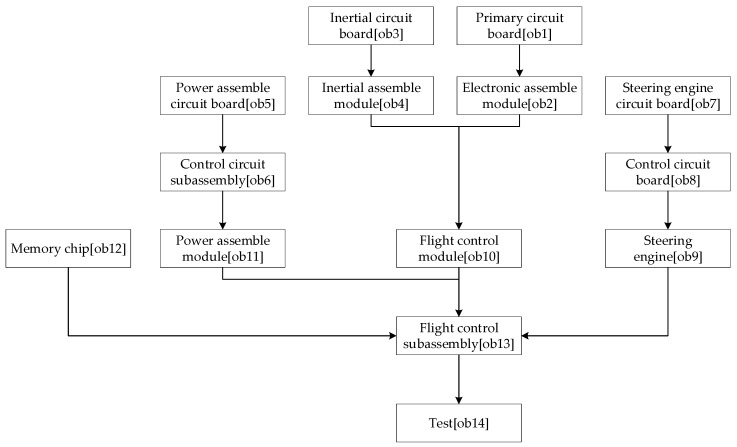
Job unit division of the system.

**Figure 3:**
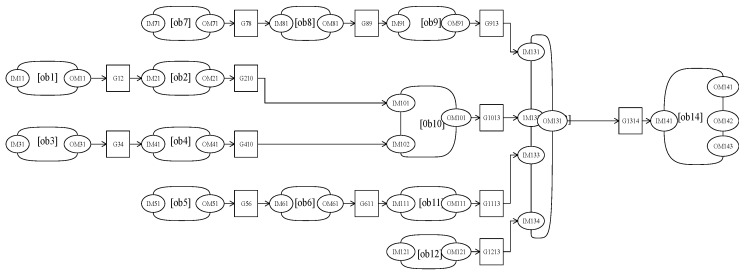
The OOPN net of the assembly process.

**Figure 4:**
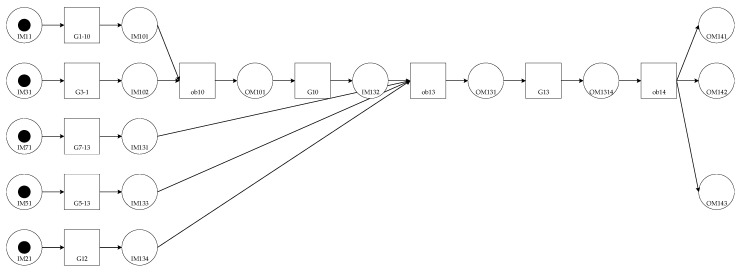
The OOPN with tokens.

**Figure 5:**
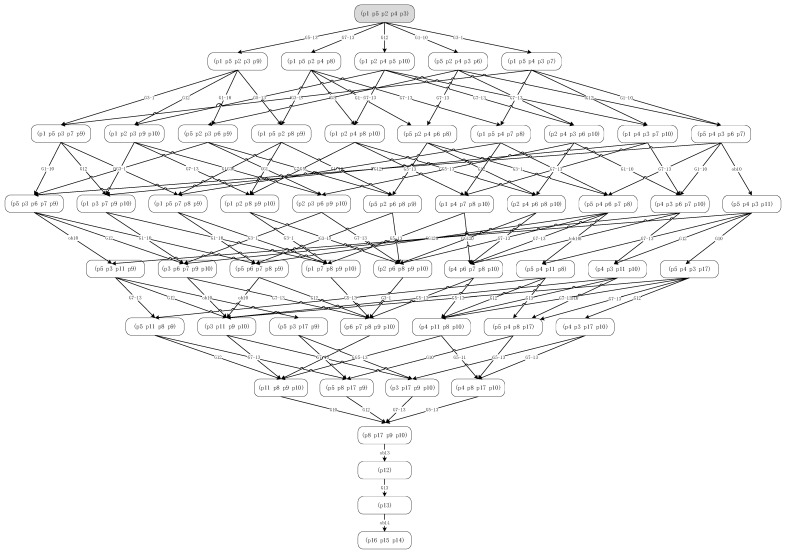
The reachability tree of this assembly process.

1. Modeling and analysis
  As shown in Figure 2, the assembly process is divided into units.
  The OOPN of the assembly process is demonstrated in Figure 2. Details about the inputs and outputs are shown in the Table 1 and Figure 3 below.
  As can be seen, no conflict shows in the Petri net, which means conflict in the assembly process does not exist.
2. Evaluating the uncertainty in the assembly process
  The initial *M*_0_ in this case is {IM11 = 1, IM31 = 1, IM51 = 1, IM71 = 1, IM121 = 1}, suggesting that when the system works, the primary circuit board, inertial circuit board, steering engine circuit board, power assembly circuit board, and memory chip have been prepared.
  All reachable markings *R*(*M*_0_) of this OOPN in Figure 4 are as follows: 51 state sets, and 10 transforms. The *R*(*M*_0_) matrix is transposed into the following form: the rows are p1 to p17 and the column is *M*_0_ to M51, from left to right the places are: (IM11, IM31, IM71, IM51, IM21, IM101, IM102, IM131, IM133, IM134, OM101, OM131, IM1314, OM143, OM142, OM141, IM132). Considering the specific values of transition firing rate into consideration, the reachability tree which built by the reachability sets during the process is shown as a semi-Markov chain in Figure 5. The reachability tree is drawn by the absorbing states during this process until reaching the steady states:
  (28) $$R{\left(M_{0}\right)}^{T}=\left[\begin{array}{ccccccccccccccccc} 1 & 1 & 1 & 1 & 1 & 0 & 0 & 0 & 0 & 0 & 0 & 0 & 0 & 0 & 0 & 0 & 0\\ 1 & 1 & 1 & 0 & 1 & 0 & 0 & 0 & 1 & 0 & 0 & 0 & 0 & 0 & 0 & 0 & 0\\ 1 & 1 & 0 & 1 & 1 & 0 & 0 & 1 & 0 & 0 & 0 & 0 & 0 & 0 & 0 & 0 & 0\\ 1 & 1 & 1 & 1 & 0 & 0 & 0 & 0 & 0 & 1 & 0 & 0 & 0 & 0 & 0 & 0 & 0\\ 0 & 1 & 1 & 1 & 1 & 1 & 0 & 0 & 0 & 0 & 0 & 0 & 0 & 0 & 0 & 0 & 0\\ 1 & 0 & 1 & 1 & 1 & 0 & 1 & 0 & 0 & 0 & 0 & 0 & 0 & 0 & 0 & 0 & 0\\ & & & & & & & & \vdots & & & & & & & & \end{array}\right]$$
  Calculating the steady-state probability vector is possible, and the solution of this chain, the steady-state probability vector, is given by:
  (29) $$\eta T=[\begin{array}{l} 0.2217,0.1736,0.173,0.163,0.0232,0.0219,0.0122,0.0142,0.0124,\\ 0.0191,0.0289,0.00671,0.00782,0.03392,0.05319,0.00893,0.02623 \end{array}]$$
  Subsequently, entropy of the network can be expressed by:
  (30) $$H(OOPN)=-\sum \eta_{i}\log_{2}\eta_{i}=3.22408$$
  The maximum entropy is the limit and, in this process, is log217 = 4.087463. The normalization with maximum entropy makes the uncertainty index a dimensionless quantity which is appropriate for the comparison of models with a different number of reachable marking [30]. The uncertainty index for this case is determined by the formula:
  (31) $$H(OOPN)/log_{2}\left|R\left(M_{0}\right)\right|,\;3.22408/4.087463=0.788772$$

The result can be approximately interpreted as the situation where the uncertainty of this case reaches 78.87% of the maximum. 

**Figure 6:**
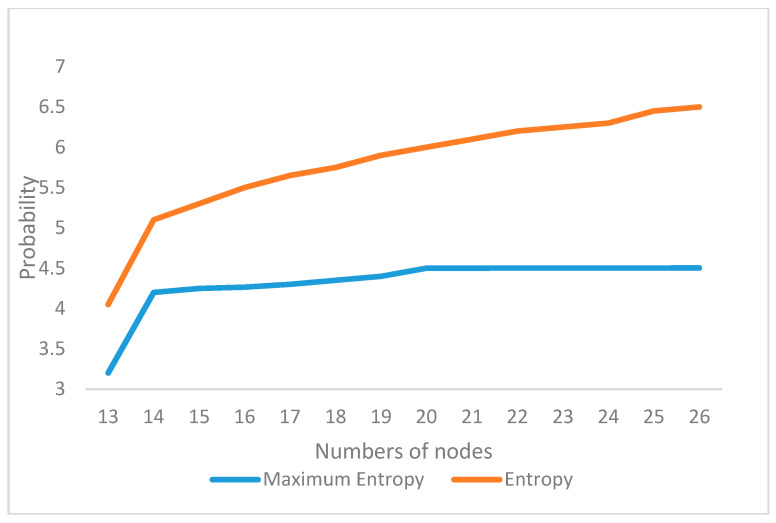
Entropy and maximum entropy.

**Figure 7:**
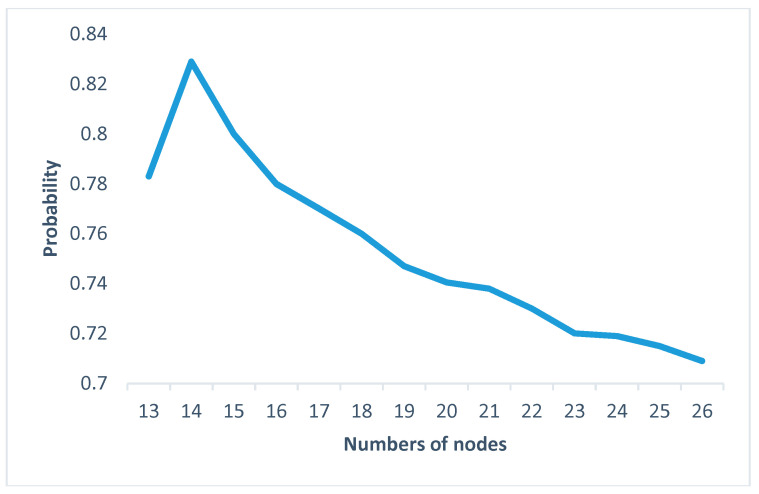
Uncertainty changing.

**Figure 8:**
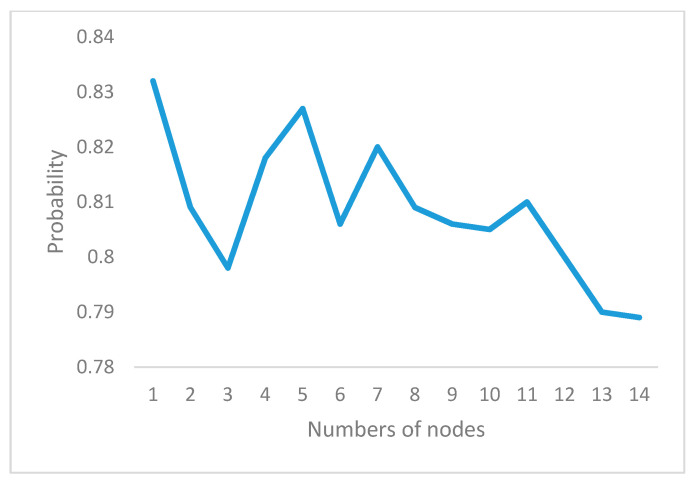
Testing location vs. uncertainty.

The uncertainty is analyzed as a response to changes in the parameters of the OOPN. The number of tokens in the initial marking is ensured. In the actual assembly process, some tests in each process should be conducted so that the places in the OOPN and the defect stream function can be developed. In the following paragraph, an example is presented to demonstrate the development of the uncertainty concerning some settings of different places and various values of parameter *λ*. In Figure 6 and Figure 7 *X*-axis represents the number of screenings. For example, the number of 13 represents the screening nodes after ob1, and in Figure 8 *X*-axis represents ob1 to ob13, which has been tagged in Figure 5. The evolution of entropy and maximum entropy are shown in Figure 7, and Figure 8 expresses the uncertainty of the process.

The location of testing is added from ob1 to ob13, suggesting that the filter points are added from the first assembly point without changing the assembly order, that is, a detection point is added to detect ob1, and two detection points, ob1 and ob2, are added. Observing the overall trend, the number of places and the uncertainty index play a decisive role in the whole process, indicating that the increasing number of places (detection method adopted is nondestructive testing) decreases the uncertainty index of the OOPN. As shown in Figure 8, a turning point appears when the two test points are added into the assembly process. The steady-state probability obtained by the OOPN, which is built without testing, is employed to analyze the cause of the turning point. In the ob1 state, the steady-state probability of 1 is lower than that of the other, which is the whole assembly process; hence, ob1 is the relatively weak link.

To verify the above conclusions, the experiment analysis is described below:

Only one detection point is added and placed in ob1 to ob13, after which the uncertainty of the assembly process after the addition of the detection point is calculated. The horizontal axis represents where the detection points are added, and the vertical axis represents the degree of influence on the result after detection.

In other words, according to the stead-state probability, the weak link can be found in the assembly process, therefore, detection location will be assigned. 

For the uniformity of the stationary probability distribution between markings, the declining uncertainty can be verified in the analysis of the distribution of the individual sets of all reachable markings. Here, λ exerts influence on the steady probability. In this study, the uncertainty is decreased as well as the defect stream in the process. 

**Figure 9:**
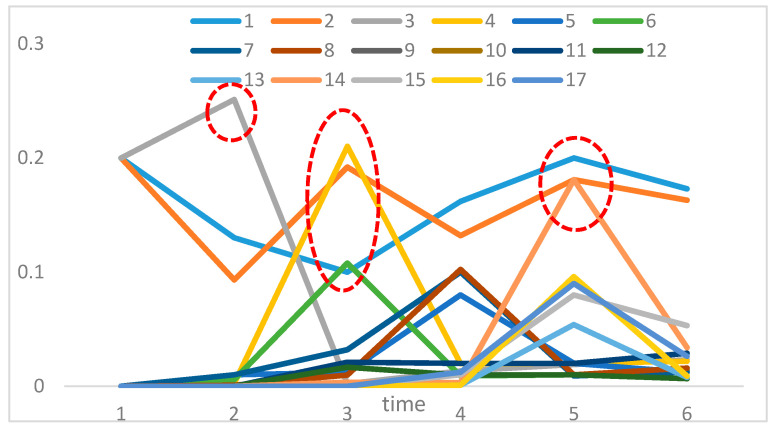
State probability.

The probability of each step of 17 nodes is as follows: the x-coordinate refers to the time and the y-coordinate represents the transition probability. Different colors represent different nodes, and Figure 9 shows the trend of state probability of each node under the assembly process. According to the research mentioned in Section 2.3, the state probability is proportional to the inefficiencies of the product under the current process; therefore, a high state probability corresponds to a high failure rate. As shown in Figure 9, the point of the red circle represents the point of efficiency loss in the process or the failure of the entire assembly process. For each curve, the instability degree is low and the process must then be re-focused.

The following schemes, based on the above analysis are proposed in Table 2:

In scheme 1, screening is performed. In scheme 2, each process was tested; scheme 3 is proposed by engineering practice (the processes of screening are 1, 2, 3, 4, 5, 7, 9, 10, 11, and 13). Combined with Figure 7, Figure 8 and Figure 9 the new screening scheme is scheme 4 (the processes of screening are 1, 2, 3, 4, 6, and 14). The results are as follows:

Obviously, according to the analysis, the optimization scheme, scheme 4 is not only uncertain, but also high in the qualified rate. Although the uncertainty was greatly reduced in scheme 2, the improvement of the rejection rate caused by too much screening, resulting in a decrease in the yield. It is very important to evaluate the effective screening position according to the overall uncertainty evaluation. 

Components within the potential defects caused by the assembly process are to inspire and lead to a relatively low failure rate of the pull level component of the initial growth stage; the main reason is that the phase products do not meet the fatigue limit and are not stimulated as apparent defects. However, the inefficiencies started to dramatically rise, and the components were exhausted under the long-term stress of the assembly process, bringing about the emergence of dominant defects. If the potential defects can be eliminated early by screening, the high failure stage can be avoided and the risk can be reduced in the meanwhile.

With the type of analysis, various assumptions on the assembly process can be made and the type of process can be evaluated when various parameters of the model are adjusted. The aim of this paper is to put forward an efficient approach to calculate uncertainties in the assembly process.

## 5. Discussion

We can easily notice that the uncertainty of the Petri net is related to the steady-state probability of each place. Steady-state refers to the probability means the possibility of maintaining a relatively stable state of development. Given that the assembly process is constituted by multiple layers, more complication in the assembly process corresponds to greater interaction processes. In consideration of the case, the component level steady-state probability is high, whereas the system-level steady-state probability is low.

The same structure exists in the assembly process; by increasing the part-level steady-state probability, the overall assembly process can be achieved. According to the above analysis, the following comparative test is conducted and the results are shown in Table 3.

**Figure 10:**
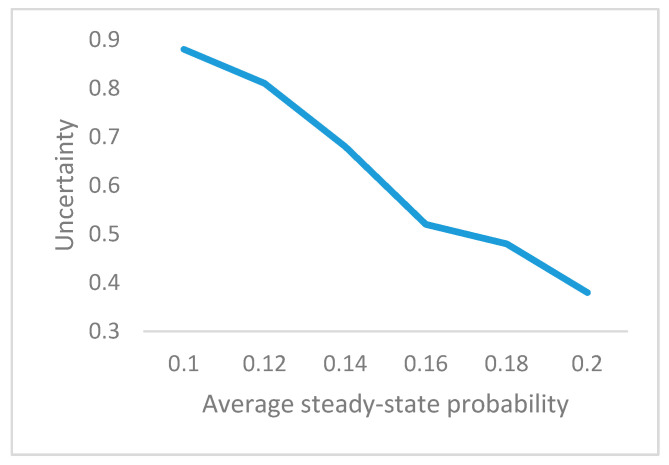
The result of the discussion.

1. Improving the process to reduce or improve the changes of defect during the assembly process;
2. Obtaining the reachability marking and calculating the steady-state probability; and
3. Measuring the uncertainty of the system;

Based on the above five sets of data, the steady-state probability of the five components for these five-group experiment is averaged. The relationship between the mean value of the component-level steady-state probability and the uncertainty is shown in the Figure 10.

By reducing the defect change and improving the low-level assembly of the steady-state probability, the uncertainty of the assembly process can be decreased. In other words, ensuring the stability of the low-level assembly process is particularly important for the entire assembly process. Therefore, the uncertainty of the system gradually decreases with the decreasing component level uncertainty.

In actual production, an assembly line is required to be assembled for various batches of products because they have different assembly processes. With this method, the assembly processes are analyzed and the weak points are located, thus allowing managers to devise a comprehensive test strategy.

## 6. Summary and Conclusions

In the present study, the multistage assembly process was analyzed and the effect of defect changes on product quality during the assembly process was investigated. An uncertainty evaluation model was developed for the multistage assembly process based on an enhanced OOPN and Shannon entropy. By simplifying the concrete assembly process to the abstract Petri net structure, the uncertainty of the assembly process was examined. An OOPN model was proposed to analyze the assembly process. Based on the historical data, the probability density function show that the defect changing process from latent to dominant upon the time process was fitted. This paper presented an application case in which the activation function in Petri net was substituted with this fitting function. The definition of entropy in physics was applied to characterize the uncertainty of the model and evaluate the assembly process. 

Finally, a flight control module assembly process was analyzed as a case study to illustrate the effectiveness of the presented approach. The results showed that the uncertainty in this case reached 78.8772% of the maximum. Subsequent analysis of subsystem uncertainty was conducted to identify weak points to modify or standardize the loading distribution in different nodes, which could reduce the uncertainty of the assembly process.

## Figures and Tables

**Table 1:** The input and output information of the Petri net.

HOOPN Objects	Input Place	Output Place
Ob1 (Primary circuit board)	IM11: start to set the primary circuit board	OM11: complete the circuit board assembly
Ob2 (Electronic assembly module)	IM21: start to assemble the electronic module	OM21: complete the electronic module
Ob3 (Inertial circuit board)	IM31: start to set the inertial circuit board	OM31: complete the inertial circuit board
Ob4 (Inertial assembly module)	IM41: assemble the inertial module	OM41: complete the inertial module
Ob5 (Power assembly circuit board)	IM51: set the power circuit board	OM51: this step is finished
Ob6 (Control circuit subassembly)	IM61: fit the subassembly of control circuit	OM61: complete this fitting
Ob7 (Steering engine circuit board)	IM71: start to fit the steering engine circuit board	OM71: complete the steering engine circuit board
Ob8 (Control circuit board)	IM81: fit the control circuit board out	OM81: finished fitting
Ob9 (Steering engine)	IM91: start to fit the steering engine together	OM91: complete the steering engine
Ob10 (Flight control subassembly)	IM101: get the electronic assembly module IM102: get the inertial assembly module	OM10: complete the flight control subassembly
Ob11 (Power assemble module)	IM111: set the power module	OM111: complete the power module
Ob12 (Memory chip)	IM121: get and test the memory chip	OM121: complete
Ob13 (Flight control module)	IM131: get the memory chip IM132: get the power module IM133: get the flight control subassembly IM134: get the steering engine	OM131: complete the flight control module
Ob14 (Test)	IM141: flight control module	OM141: qualified OM142: below the mark (repairing) OM143: scrap

**Table 2:** The result of different schemes.

Evaluation	Process Scheme			
	Scheme 1	Scheme 2	Scheme 3	Scheme 4
Uncertainty	78.8772%	16.89%	34.885%	15.367%
Yield	34.2%	31.8%	51.3%	81.6%

**Table 3:** The results of the analysis.

No.	Process Level	Steady-State Probability	Entropy	Uncertainty
1	−	*ηT* = [0.2217,…0.02623]	3.22408	78.87%
2	↑	*ηT* = [0.302,…0.00012]	2.33529	57.13%
3	↑	*ηT* = [0.51,…0.00001]	2.0158	49.31%
4	↓	*ηT* = [0.103,…0.02]	3.5941	87.93%
5	↓	*ηT* = [0.09,…0.1]	3.68	90.01%
